# Performance evaluation of mNGS in pathogen diagnosis of skin and soft tissue infections and its optimization effect on antibiotic decision-making

**DOI:** 10.3389/fcimb.2026.1771148

**Published:** 2026-04-10

**Authors:** Xuyang Li, Jiaao Fang, Dongqin Li, Bingjie Cai, Jie Yin, Yunpeng Zheng, Guangwen Yin

**Affiliations:** 1 Department of Dermatology, First Affiliated Hospital of Zhengzhou University, Zhengzhou, Henan, China; 2 Department of Oncology, First Affiliated Hospital of Zhengzhou University, Zhengzhou, Henan, China

**Keywords:** culture, medication adjustment, mNGS, multiple infections, SSTIs

## Abstract

**Background:**

Skin and soft tissue infections (SSTIs), often caused by polymicrobial pathogens, pose diagnostic challenges due to the limitations of conventional methods, including low sensitivity and prolonged turnaround time. This diagnostic gap has perpetuated empirical antibiotic use in clinical practice. Metagenomic next-generation sequencing (mNGS), with its unbiased pathogen detection capability, offers a transformative approach for rapid and precise microbial identification in SSTIs.

**Objective:**

To evaluate the clinical utility of mNGS compared to conventional microbiological testing in guiding antibiotic stewardship for complex SSTIs.

**Methods:**

A retrospective cohort study was conducted at the First Affiliated Hospital of Zhengzhou University from April 2023 to May 2025, enrolling 69 patients with clinically diagnosed complex SSTIs. All patients underwent concurrent mNGS testing, conventional bacterial culture, and pathological examination. The diagnostic performance of mNGS was systematically compared with culture methods, with emphasis on culture-negative cases and polymicrobial infections. The impact of mNGS-guided antibiotic adjustments was assessed.

**Results:**

mNGS demonstrated significantly higher pathogen detection rates than conventional culture (P < 0.001), with a concordance of 37.5% between the two methods. Among 24 culture-negative patients, mNGS identified pathogens in 20 cases (83.3% detection rate). For polymicrobial infections (n = 20), culture detected pathogens in only 2 cases, whereas mNGS successfully identified multiple pathogens in the majority. Antibiotic therapy was adjusted based on mNGS results in 11.9% (8/69) of patients.

**Conclusion:**

mNGS substantially improves pathogen detection in complex SSTIs compared to conventional methods. Beyond diagnostic accuracy, its clinical value lies in enabling targeted antibiotic therapy, thereby optimizing antimicrobial stewardship and potentially reducing healthcare costs.

## Introduction

Skin and soft tissue infections (SSTIs) represent a prevalent clinical entity in dermatology and infectious disease practice, characterized by an extraordinarily diverse microbial spectrum encompassing bacteria, fungi, viruses, and parasites (Stevens et al., 2014). Clinically, these infections manifest with non-specific presentations, ranging from acute suppurative lesions, ulcerations, and nodules to chronic recalcitrant skin manifestations (Ki and Rotstein, 2008). Pathogenesis is intricately linked to the disruption of cutaneous barrier integrity, dysbiosis of local microbiota, and impairment of host immune defenses, with a subset of patients developing polymicrobial infections or systemic complications (Dryden, 2009).

The conventional diagnostic paradigm for skin and soft tissue infections (SSTIs) predominantly relies on histopathological examination, bacterial culture, smear microscopy, PCR-based assays, and serological testing (Esposito et al., 2016). Among these, histopathological analysis serves as the cornerstone for definitive diagnosis, while bacterial culture remains indispensable for etiological confirmation and antimicrobial susceptibility profiling (Sanchez-Romero et al., 2019). However, these traditional methodologies confront mounting technical limitations. Microbial culture, as the principal diagnostic approach, suffers from prolonged turnaround times and results that are highly contingent upon microbial viability and sample integrity, resulting in markedly reduced sensitivity for anaerobes, fastidious organisms, and rare pathogens (Sanchez-Romero et al., 2019). Meanwhile, PCR and serological assays, despite their commendable specificity, are inherently limited by their targeted nature, rendering them ineffective for identifying novel pathogens or co-infections with multiple microbial species (Liu et al., 2011). The combined diagnostic constraints of sensitivity limitations and temporal delays have precipitated widespread empirical, broad-spectrum antimicrobial therapy in clinical practice, exacerbating antibiotic resistance risks and potentially compromising patient outcomes (Claxton et al., 2020).

Metagenomic next-generation sequencing (mNGS) enables unbiased, high-throughput sequencing of all nucleic acid sequences in clinical samples without requiring culture or prior target assumptions (Schlaberg et al., 2017). Through bioinformatic alignment, this method can identify a broad spectrum of microbial taxa, including bacteria, fungi, viruses, parasites, and atypical pathogens (Schlaberg et al., 2017). Compared to conventional methods, mNGS demonstrates significantly enhanced detection sensitivity and pathogen coverage, particularly excelling in identifying rare pathogens, polymicrobial infections, and samples from post-antimicrobial therapy cases (Han, 2022). Critically, its turnaround time can be reduced to 24–48 hours, providing robust technical support for rapid etiological diagnosis of challenging and life-threatening infections (Han, 2022).

However, as mNGS technology becomes more widely adopted and clinical experience accumulates, the prevailing challenges have shifted from “whether detection is possible” to “how to interpret and apply the vast information generated”. Against the backdrop of escalating global antibiotic resistance, leveraging mNGS data transcends mere pathogen identification; it is instrumental in guiding precision therapy and promoting rational antimicrobial use (Keck et al., 2024; Ma et al., 2025; Zou et al., 2025). Building upon prior research, this study analyzed clinical data from 69 patients with suspected complex skin infections. We systematically compared mNGS with conventional laboratory testing across three dimensions: pathogen detection rates, diagnostic timeliness, and microbial diversity coverage. Furthermore, we meticulously examined antibiotic therapy adjustments before and after mNGS reporting to quantify its actual impact on clinical decision-making and patient outcomes.

## Methods

### Study design and cohort selection

This retrospective observational study analyzed clinical data from adult patients (≥18 years) diagnosed with complicated skin and soft-tissue infections (cSSTIs) at the First Affiliated Hospital of Zhengzhou University between April 2023 and May 2025. The study cohort was defined by clinically confirmed cSSTIs, including necrotizing fasciitis, deep-seated abscesses, surgical-site infections, and infections in immunocompromised hosts. Inclusion criteria comprised: 1, Age ≥18 years; 2, Confirmed diagnosis of one or more cSSTI subtypes; 3, Availability of complete clinical documentation. Exclusion criteria included: 1, Concurrent active extra-cutaneous infections;2, Pregnancy or lactation status; 3, Incomplete medical records. After systematic screening, 69 patients meeting eligibility criteria were included in final analysis. The study protocol received ethical approval from the institutional review board of the First Affiliated Hospital of Zhengzhou University (approval no. 2025-KY-1377).

All clinical data were extracted from institutionally maintained electronic health records and laboratory information systems. Personally identifiable information was systematically removed during data processing. The study was conducted in full compliance with the ethical principles of the Declaration of Helsinki. No additional diagnostic procedures or interventions were performed for research purposes, ensuring no deviation from standard clinical care protocols.

### Specimen collection

The sampling protocol was tailored to the anatomical site of skin and soft-tissue involvement. Under aseptic conditions, clinical physicians collected epidermal cells or exudates from the lesion site, including purulent material, exudate, wound swabs, or tissue biopsies. All specimens were processed in parallel for histopathological evaluation, conventional bacterial culture, and metagenomic next-generation sequencing (mNGS).

For histopathological analysis, tissue samples were immediately fixed in formalin and processed into sections for microscopic examination. Bacterial culture specimens were transported to the microbiology laboratory for inoculation and incubation under standard conditions. mNGS-compatible samples were cryopreserved at ≤−80 °C within 2 hours of collection and shipped to the sequencing facility for nucleic acid extraction and high-throughput sequencing within 24 hours.

### Bacterial culture

All clinical specimens were transported to the clinical microbiology laboratory within 2 hours post-collection. Based on sample type (e.g., purulent material, wound swabs, skin tissue, or exudate), standardized pre-processing was performed, followed by inoculation onto appropriate culture media: blood agar plates for general aerobic bacteria, MacConkey agar for Gram-negative bacilli, chocolate agar for fastidious organisms, and selective anaerobic media when indicated. Fungal culture was conducted using Sabouraud dextrose agar when clinically warranted.

Observe the colony morphology after cultivation and perform Gram staining. Suspected bacterial colonies were purified and identified as the main bacterial species; For strains that cannot be clearly identified by mass spectrometry, automated biochemical identification systems or traditional biochemical experiments are used to complete the identification. Fungi need to be interpreted based on their cultivation characteristics and microscopic morphology.

### Laboratory parameter collection and analysis

All laboratory data were extracted from electronic medical records at admission, encompassing:

1, Complete blood count (CBC): White blood cell count (WBC), red blood cell count (RBC), hemoglobin (Hb), platelet count, and differential counts (absolute values and percentages of neutrophils, lymphocytes, monocytes, eosinophils, and basophils). 2, Inflammatory and infection markers: C-reactive protein (CRP), procalcitonin (PCT), and D-dimer. 3, Additional biochemical and coagulation parameters: As clinically indicated (e.g., liver function tests, renal function tests, coagulation profiles). All laboratory assays were performed in the Clinical Laboratory Department of the First Affiliated Hospital of Zhengzhou University following standardized operating procedures (SOPs). Statistical analysis: Continuous variables were presented as median (interquartile range, IQR: Q1–Q3). Categorical variables were expressed as counts (percentages). These parameters were utilized for between-group comparisons and correlation analyses in subsequent statistical modeling.

### Metagenomic next-generation sequencing protocol

Extract nucleic acids from clinical specimens according to the operating procedures of the reagent kit and take a certain amount of total nucleic acids for ultrasonic or enzyme sectioning processing. Complete the library building steps of end repair, 3 ‘end A addition, and connector connection in sequence according to the requirements of the mNGS library building reagent kit. Subsequently, the library was amplified, and the product quality was controlled. Qualified libraries undergo denaturation to form single stranded DNA, which is further cyclized to generate multi copy DNA nanospheres (DNBs) using rolling ring replication technology. The prepared DNBs were loaded into the holes of the sequencing chip using high-density DNA nanochip technology, and high-throughput sequencing was performed using co probe anchored polymerization (cPAS) technology. This study chose the double ended 50bp (PE50) sequencing mode and completed the sequencing using the DNBSEQ-200 platform (MGI, China).

The raw data obtained from sequencing is filtered by quality control to remove low-quality reads and adapter contamination, and then clean data is obtained. Subsequently, the host sequence of clean data was removed and aligned to a standard microbial reference database for species annotation. Combined with the analysis results after filtering the laboratory background bacteria, the possible pathogenic microbial species and their sequencing abundance in the sample were finally obtained.

### Pathogen identification criteria

In the diagnostic framework for skin and soft tissue infections (SSTIs), histopathological examination serves as the diagnostic reference standard for confirming infection characteristics and defining pathological features — particularly in cases of deep fungal infections, granulomatous inflammation, or clinically ambiguous presentations where microbiological or molecular assays alone provide insufficient diagnostic clarity. Histopathological interpretation, integrated with clinical manifestations and corresponding microbiological findings, provides definitive evidence for final diagnosis.

### Data collection on antimicrobial medication

A comprehensive record of anti-infective medication regimens was compiled for all enrolled patients, spanning from initial presentation through the follow-up period. Documentation encompassed antibacterial agents, systemic and topical antifungal preparations, and combination therapy protocols. Parameters included drug classification, treatment duration, number of concomitant antimicrobial classes (categorized as monotherapy, dual therapy, triple therapy, or ≥4-drug combinations). Documentation of associated adverse drug reactions was systematically performed. Furthermore, treatment modifications guided by either mNGS or conventional culture results were specifically annotated to assess the impact of different diagnostic modalities on clinical decision-making. All medication data were sourced from institutional electronic health records (EHR) and inpatient pharmacy databases.

### Statistical analysis

All statistical analyses and data visualization were performed using R software (version 4.3). Based on mNGS findings, patients were stratified into positive and negative cohorts for comparative analysis of demographic characteristics, clinical presentation, and laboratory parameters.

Continuous variables were assessed for normality using the Shapiro-Wilk test. Non-normally distributed data are presented as median with interquartile range (IQR; Q1, Q3), and between-group comparisons were conducted using the Wilcoxon rank-sum test. Categorical variables are summarized as counts (percentages), with differences evaluated by Fisher’s exact test. Statistical significance was defined as a two-tailed P value < 0.05. All analytical procedures were rigorously documented to ensure reproducibility.

## Results

### Patient demographic and clinical characteristics

Following the exclusion of specimens with incomplete mNGS data, the cohort was stratified into an mNGS-negative group (Group N, n = 10) and an mNGS-positive group (Group P, n = 53). Comparative analysis revealed no statistically significant differences in gender distribution or median age between the two groups, and conventional pathogen culture results were largely concordant (**Table 1**).

**Table 1:** mNGS and culture consistency.

	mNGS_positive	mNGS_negative	Total
Culture_positive	8	1	9
Culture_negative	24	7	31
Total	32	8	40

Laboratory Parameter Comparisons Significant intergroup disparities were observed across multiple inflammatory and hematological indices. Specifically, Group P demonstrated markedly lower levels of white blood cell count (WBC, p = 0.002), platelet count (PLT, p = 0.026), and absolute neutrophil count (Neut, p = 0.003) compared to Group N. Monocyte (Mono, p = 0.013) and eosinophil (Eos, p = 0.010) counts were also significantly reduced in the mNGS-positive cohort. Procalcitonin (PCT, p = 0.028) concentrations were substantially diminished in Group P relative to Group N.

Parameters with No Significant Differences No statistically significant differences were detected between groups for the remaining parameters, including red blood cell count, hemoglobin level, lymphocyte count, basophil count, C-reactive protein (CRP), and D-dimer levels.

### Concordance analysis between mNGS and blood culture

Among the 32 cases with positive mNGS results, only 8 exhibited concurrent positivity by blood culture, while 24 were culture-negative. Overall concordance between the two methods was 37.5%. Of the 9 patients with positive blood culture, mNGS correctly identified 8 (true positive), while 1 returned negative (false negative). Conversely, among 31 blood culture-negative cases, mNGS produced positive signals in 24 instances—corresponding to a false-positive rate (FPR) of 77.42%, compared to a false-negative rate (FNR) of 11.11% (**Table 2**).

**Table 2:** Baseline of mNGS negative and positive patients.

Characteristic	Negative N=10	Positive N=53	p-value
Gender			0.5
Female	4 (40%)	30 (57%)	
Male	6 (60%)	23 (43%)	
Age	53 (18, 61)	60 (52, 70)	0.2
Culture			>0.9
Negative	1 (100%)	20 (71%)	
Positive	0 (0%)	8 (29%)	
Unknown	9	25	
WBC	8.33 (7.04, 12.05)	5.70 (4.77, 7.25)	0.002
RBC	4.56 (4.15, 5.06)	4.41 (4.03, 4.78)	0.4
Hbg	132 (126, 157)	130 (123, 138)	0.6
PLT	325 (224, 384)	227 (199, 261)	0.026
Neut	5.18 (4.47, 7.87)	3.77 (2.82, 4.38)	0.003
Lymph	2.33 (1.08, 3.12)	1.68 (1.33, 2.17)	0.3
Mono	0.54 (0.42, 0.75)	0.37 (0.31, 0.45)	0.013
Eos	0.04 (0.01, 0.06)	0.10 (0.05, 0.15)	0.010
Baso	0.015 (0.010, 0.040)	0.020 (0.020, 0.040)	0.3
CRP(mg/L)	8 (2, 64)	2 (1, 5)	0.10
PCT(ng/mL)	0.034 (0.020, 0.046)	0.020 (0.020, 0.028)	0.028
D.Dimer(mg/L)	0.28 (0.13, 0.43)	0.19 (0.12, 0.30)	0.3

n(%); Median (Q1;Q3).

Fisher’s exact test; Wilcoxon rank sum test.

Furthermore, mNGS successfully detected pathogens in 20 of 24 blood culture-negative cases, demonstrating its enhanced capability for pathogen identification in culture-elusive scenarios. Statistical assessment of inter-method agreement yielded a Cohen’s kappa coefficient of 0.06, indicating only slight agreement beyond chance. These findings suggest that mNGS and conventional blood culture provide complementary diagnostic information in the evaluation of complex infections.

### Diverse pathogenic spectrum identified by mNGS

**Figure 1:**
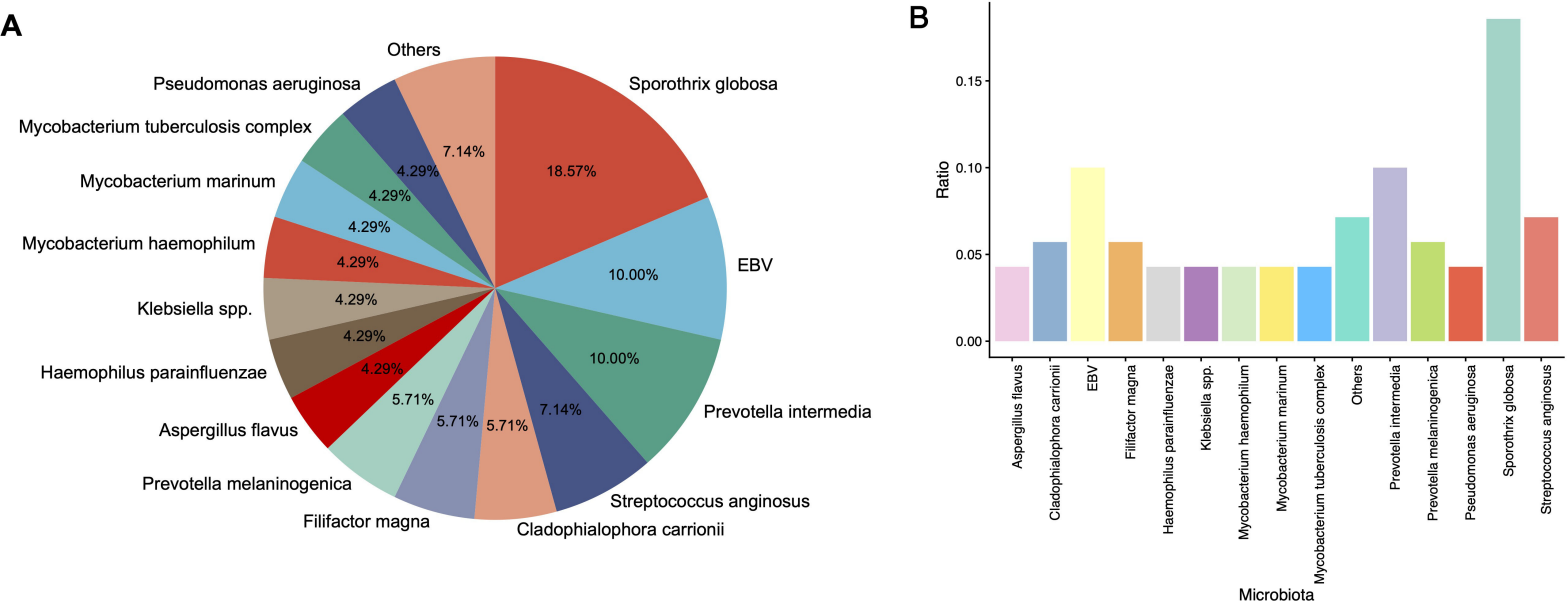
Microbial spectrum detected by mNGS technology. **(A)** The pie chart displays the top 15 detected microorganisms; **(B)** The bar chart displays the proportion of the top 15 detected microorganisms.

Metagenomic next-generation sequencing (mNGS) analysis revealed a broad array of pathogenic microorganisms, demonstrating high microbial diversity in the cohort. The overall detection profile indicated Sporothrix globosa as the predominant pathogen, accounting for 18.57% of total positive results (**Figure 1**). This was followed by *Epstein-Barr virus* (10.00%) and *Prevotella intermedia* (10.00%).

Furthermore, mNGS successfully detected microorganisms that are notoriously challenging to identify through conventional culture methods. A notable example was *Cladophialophora carrionii*, a rare conditional fungal pathogen (**Figure 1**). Such pathogens, characterized by fastidious growth requirements and slow replication rates, are frequently undetected by standard microbiological cultures. By directly sequencing microbial nucleic acids, mNGS significantly enhances the detection capability for rare and difficult-to-culture pathogens.

### Supplementary diagnostic value of mNGS in culture-negative cases

**Figure 2:**
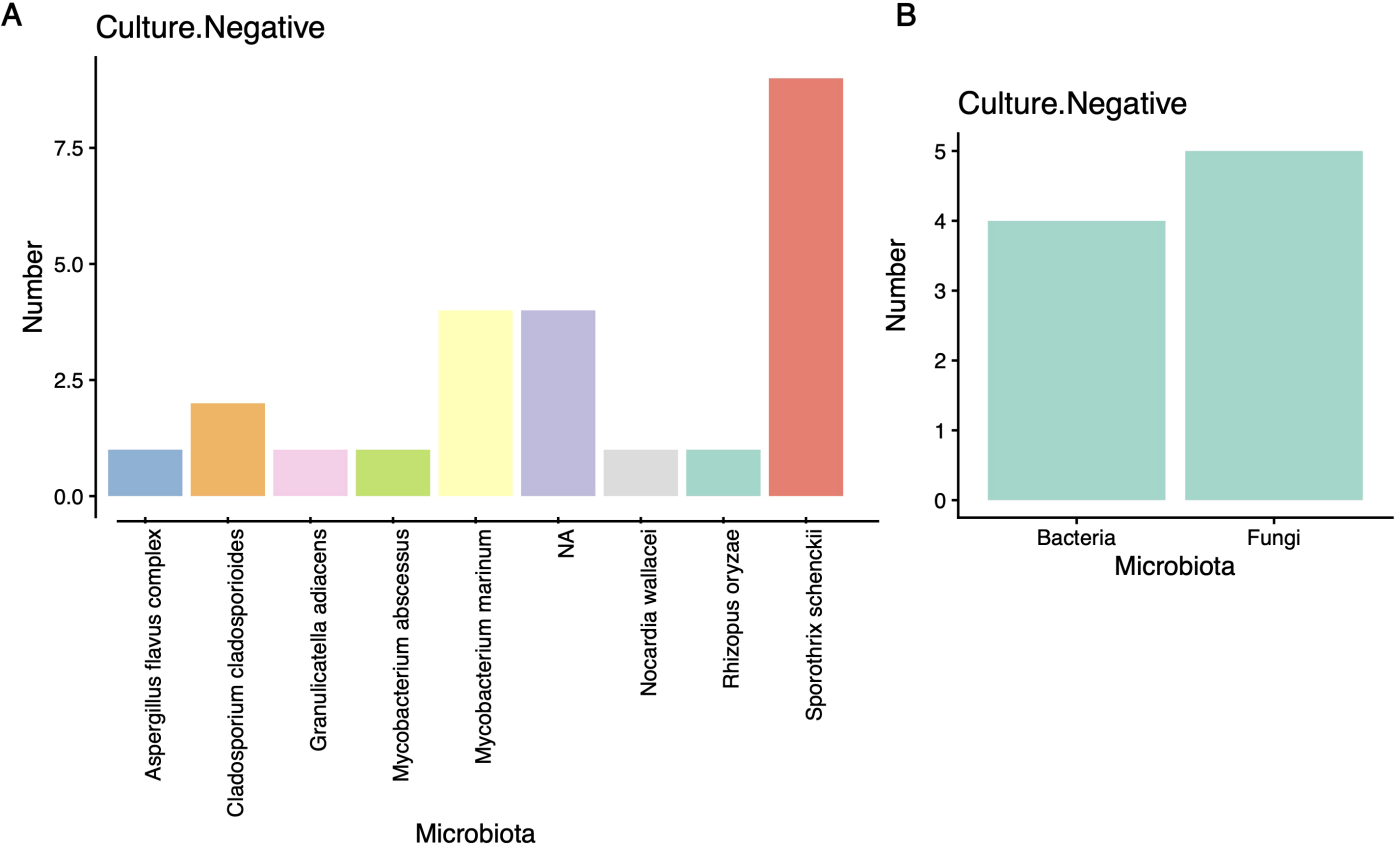
Detection of mNGS technology in culture negative specimens. **(A)** Microbial species and quantity in blood culture negative specimens; **(B)** Detecting the number of bacteria and fungi in culture negative specimens using mNGS technology.

Whereas conventional culture remains the diagnostic reference standard for microbial identification, its utility is constrained by prolonged turnaround times and the inability to cultivate fastidious organisms. Here, mNGS demonstrated significant diagnostic augmentation: Pathogens were detected in 83.3% (20/24) of culture-negative patients (**Figure 2A**). The identified microbiota comprised 4 distinct bacterial species and 5 fungal species, highlighting the method’s capacity to uncover taxonomically diverse, culture-resistant pathogens (**Figure 2B**).

### Co-infection dominates the microbiological landscape

**Figure 3:**
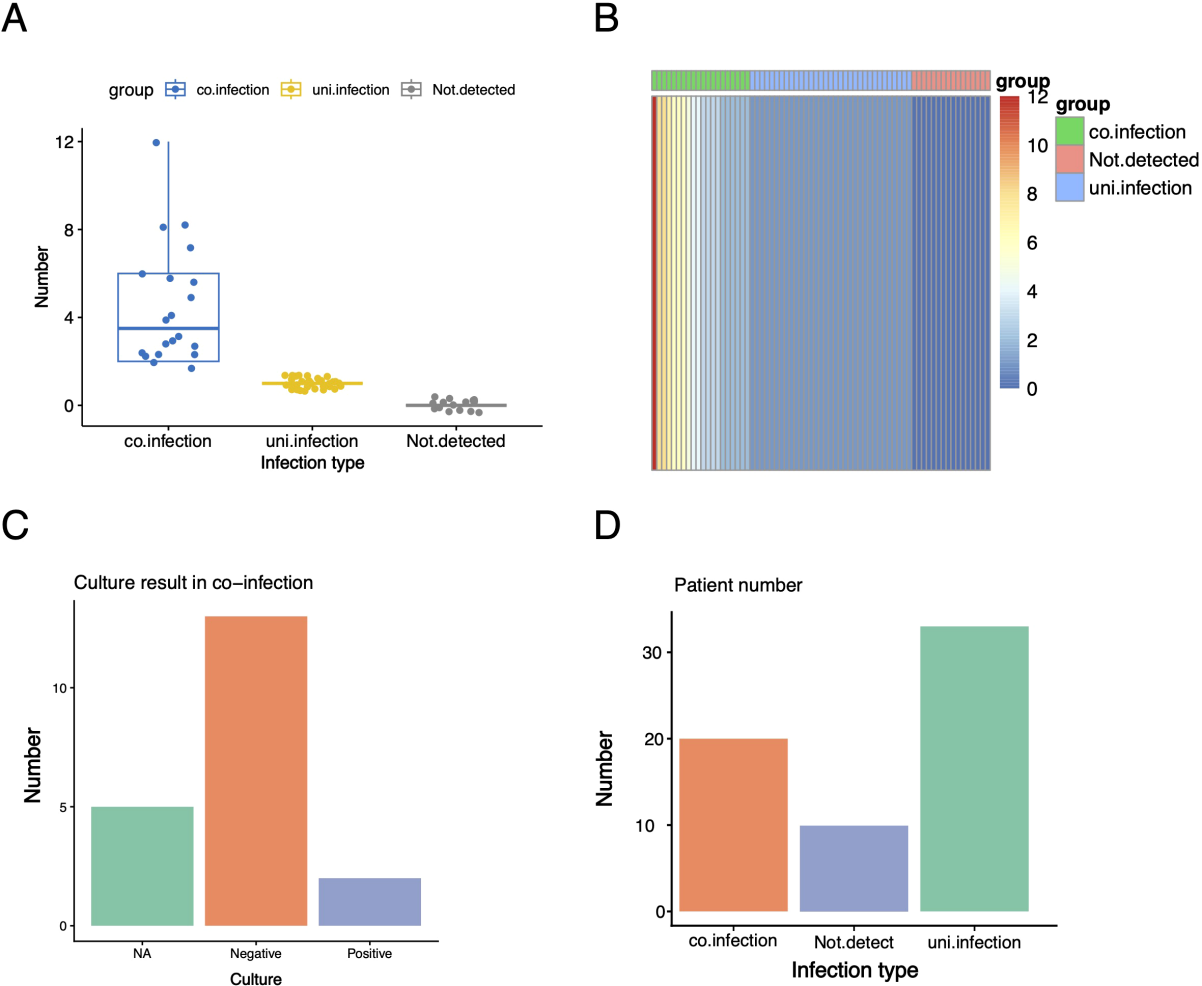
The detection capability of mNGS technology in co infected samples. **(A)** Box plot of microbial count detected in co infection, single pathogen infection, and negative patients; **(B)** Heatmap of microbial count detected in co infection, single pathogen infection, and negative patients; **(C)** The number of patients detected by culture technology in different types of infections; **(D)** The number of patients detected by mNGS technology in different types of infections.

Metagenomic next-generation sequencing (mNGS) identified polymicrobial aetiology in the majority of cases. Across the cohort, co-infection was more frequent than mono-infection, whereas no-pathogen detection was rare (**Figure 3A**). Stratification by infection category confirmed this hierarchy: mixed infections predominated, single-species infections were intermediate and negative results least common (**Figure 3B**).

Complex pathogen combinations were the rule rather than the exception. Most co-infected individuals harbored at least two distinct taxonomic groups—commonly bacterial–fungal, fungal–mycobacterial or bacterial–viral pairs—and a subset carried three or more classes concurrently (**Figure 3C**). Fungal–mycobacterial co-detection occurred most often, implying that invasive mycoses and non-tuberculous mycobacteria frequently coexist in refractory skin and soft-tissue infections. Viral reads were consistently recovered as secondary or latent signals, adding a further layer of genomic complexity. Conventional cultures yielded positive results in only a minority of these polymicrobial cases (**Figure 3D**).

### Therapeutic landscape reflects polymicrobial complexity

Complete prescription data were available for 66 of 69 participants. Thirty-eight (57.6%) received agents from two or more drug classes (**Table 3**). Exposure increased monotonically: two patients (3.0%) were prescribed no anti-infective therapy, 12 (18.2%) a single class, 14 (21.2%) two classes, 18 (27.3%) three classes, 11 (16.7%) four classes, and seven (10.6%) five or more classes (**Table 4**). Thus, combination therapy was the norm; 54.6% of the cohort received ≥3 drug categories.

**Table 3:** The overall proportion of multiple drug combination therapy in patients.

Type	Patient number
Multi drug combination therapy	38
Total	66
Multi drug ratio	57.60%

**Table 4:** Statistics of drug combination types.

Multi drug type	Patient number	Ratio
Type0	2	3.00%
Type1	12	18.20%
Type2	14	21.20%
Type3	18	27.30%
Type5	11	16.70%
Type6	7	10.60%

Type 0 represents no drug combination therapy; Type 6 represents the combination of more than 5 drugs.

**Figure 4:**
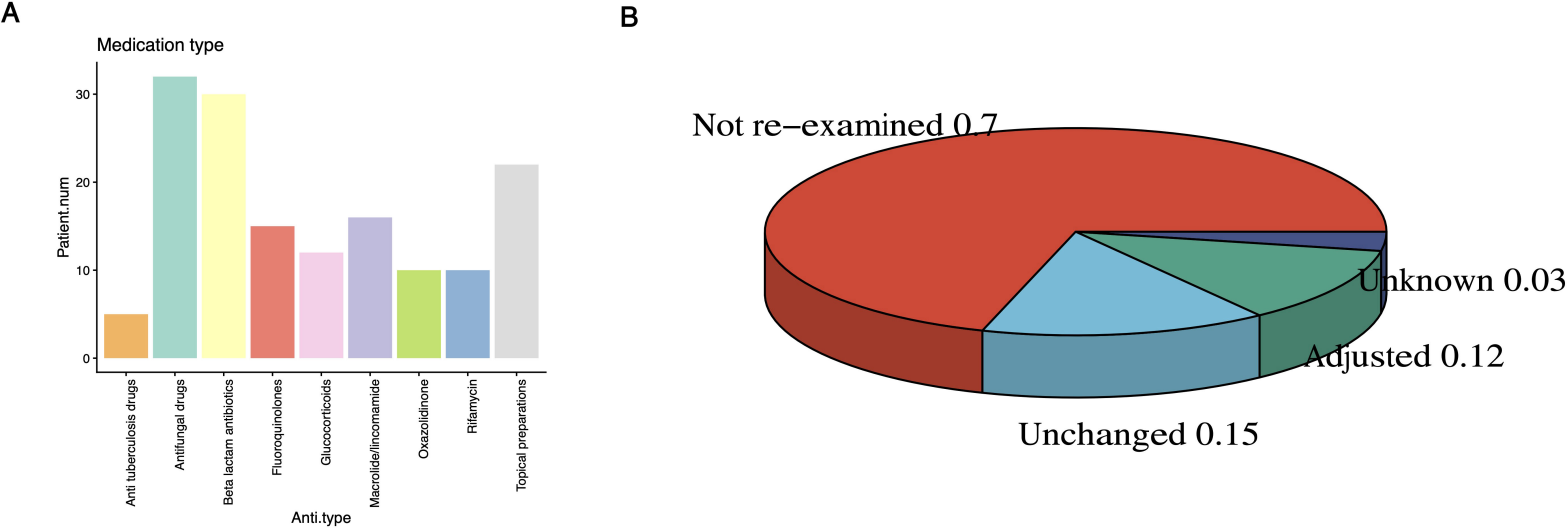
Medication use in patients with skin infections. **(A)** Bar chart showing the statistics of the number of patients with different medication types before conducting mNGS testing; **(B)** The pie chart shows the proportion of medication adjustments for patients, Not reexamined means not reexamined on time; Unknown represents missing data for unknown reasons.

Glucocorticoids were administered most frequently (n=12), indicating a need for immunomodulation or inflammation control. Linezolid and rifampicin were each used in ten patients, underscoring the importance of Gram-positive and mycobacterial coverage. Isoniazid was prescribed in four cases, mirroring mycobacterial detection by mNGS. Amphotericin B was given once, whereas topical voriconazole or polymyxin B combinations were used in four patients as local adjunctive therapy (**Figure 4A**). After mNGS testing, 8 patients (12%) came for follow-up and changed their medication, while 10 patients (15%) did not change their medication. Approximately 70% of patients did not come for follow-up, and about 44% of the patients who received medication changes during the follow-up (**Figure 4B**).

Overall, the drug-combination profile aligned with the fungal, mycobacterial and Gram-positive pathogens identified by mNGS, supporting the clinical utility of metagenomic guidance in complex skin and soft-tissue infections.

## Discussion

Metagenomic next-generation sequencing (mNGS) outperformed conventional culture across positivity rate, taxonomic breadth, polymicrobial detection and recovery of fastidious organisms. Overall positivity was 76.8%—more than double the contemporaneous culture yield and consistent with historical reports of <30% for culture-based diagnosis of SSTIs, especially after antibiotic exposure or in chronic, deep or atypical lesions. Among 24 culture-negative specimens, 20 (83.3%) were resolved by mNGS, underscoring its ability to circumvent suppression by prior antibiotics, slow growth or absent selective media. Deep-seated fungi, non-tuberculous mycobacteria (NTM) and Nocardia were detected exclusively or disproportionately by sequencing, indicating that the true burden of these taxa in SSTIs has been systematically underestimated.

The resultant pathogen profile diverged sharply from the classically described bacterial paradigm (Yeh et al., 2024; Mu et al., 2025). Fungi were identified in almost half of the cohort, with *Sporothrix globosa* predominating—far exceeding prior estimates (Gomez-Gaviria et al., 2023). NTM were detected in roughly one-quarter of cases, an order of magnitude above the <5% typically cited (Wang et al., 2022; Liu et al., 2025; Tao and Zheng, 2025). This shift is attributable to a referral bias toward refractory, chronic lesions, regional epidemiology and the superior sensitivity of mNGS for slow-growing organisms.

Polymicrobial infection of skin was common: most positive samples contained two or more taxa, and over one-third harbored three or more, with some exceeding five. Previous studies quote mixed-infection rates of 10–20% (Nimmo et al., 2020; Gouleu et al., 2024); culture identified only two positives among these cases. Thus, conventional diagnostics have long under-appreciated the complex ecological network of SSTIs. By simultaneously capturing bacterial–fungal, fungal–mycobacterial and bacterial–viral consortia, mNGS provides a high-resolution view of infection biology and offers a new perspective for understanding the pathological mechanisms and disease persistence of complex infections.

In our research, more than half of the cohort received agents from two or more anti-infective classes, and 54% were prescribed ≥3 categories, underscoring the reliance on empirical, broad-spectrum regimens for complicated SSTIs. mNGS provided an evidence base for de-escalation or redirection of therapy (Pan et al., 2025). In this study, multiple patients underwent clear drug adjustments based on mNGS results, demonstrating its significant practical value in optimizing anti infection strategies, reducing drug resistance risks, and avoiding ineffective treatments. In addition, although pathological examination cannot directly identify specific pathogens in most cases, its histological inflammatory pattern is biologically consistent with the pathogen types detected by mNGS in a considerable proportion of cases. For example, granulomatous inflammation often corresponds to chronic fungal or non-tuberculous mycobacterial infections, while suppurative inflammation is more common in bacterial infections. The mutual confirmation of the two suggests that pathology and mNGS have natural complementarity in the diagnosis of complex SSTIs.

Metagenomics cannot yet discriminate colonization from active infection, is susceptible to environmental noise, provides no phenotypic susceptibility data and remains interpreter-dependent, expensive and technically heterogeneous (Liu et al., 2025). In addition, its low specificity also suggests that it should not be used as a single diagnostic basis in clinical practice, and should be combined with pathology, imaging, clinical manifestations, and traditional microbiological testing for joint decision-making. Nevertheless, for culture-negative, refractory or suspected fungal/NTM SSTIs, mNGS supplies uniquely actionable information. Overall, the results of this study fully demonstrate that mNGS can significantly improve the pathogen detection level of complex SSTIs, reveal mixed infection patterns that have long been unrecognized by traditional methods, and promote precise treatment. Together with traditional culture and pathological examination, it constitutes the optimal combination strategy for modern SSTIs pathogen diagnosis.

## Conclusion

Metagenomics exposes the hidden polymicrobial landscape of skin and soft-tissue infections, uncovering fungi, non-tuberculous mycobacteria and multi-pathogen consortia missed by culture. By converting negative cultures into actionable pathogen maps, it enables targeted therapy, curbs empirical broad-spectrum use and limits resistance selection. Integration with conventional culture—retained for susceptibility testing—delivers a comprehensive, stewardship-compliant diagnostic framework for complex SSTIs.

## Data Availability

The original contributions presented in the study are included in the article/supplementary material. Further inquiries can be directed to the corresponding authors.
